# Falsely undetectable TSH in a euthyroid patient

**DOI:** 10.1186/s13044-020-0076-y

**Published:** 2020-01-30

**Authors:** Monireh Rahimkhani, Kiana Kazemian, Rashid Ramezani Daryasari

**Affiliations:** 1 Department of Lab Medical Sciences, Faculty of allied medical Sciences, Tehran University of Medical Sciences, Tehran, Iran; 2 Al-mahdi Clinic, Tehran University of Medical Sciences, Tehran, Iran

## Abstract

**Background:**

Measuring thyroid hormone levels is essential and helpful in the diagnosis of thyroid diseases.

**Case presentation:**

We had a patient with undetectable serum TSH level by the Siemens immunoassay, whereas by ELISA and Roche-Abott immunoassay, serum level of TSH was in the normal range.

**Conclusion:**

If the result of TSH level revealed very low or undetectable through one specific method, it should not be considered necessarily as a symptom of hyperthyroidism. It is to be requested to measure by the other methods with other devices too.

## Background

Measuring thyroid hormone levels, especially TSH is essential and helpful in the diagnosis of thyroid dysfunction or diseases. Immunoassay is currently the best way to measure hormone levels. Of course, other methods, such as ELISA and gamma counter, are also used in some laboratories.

## Case report

A 24-year-old woman attended to Al-Mahdi Clinic Lab with a request for thyroid function tests and other routine checkup tests. This patient had no history of disease or symptoms of malfunctioning of thyroid gland. Test results showed that total and free serum levels of T3 and T4 were normal, whereas TSH levels were less than 0.004 IU/ml (Normal range for adults 0.24–5.4 IU/ml). By confirming the results, Thyroid function tests were repeated by new sampling after 1 week and the same results were obtained. The method were used for detecting serum TSH level was immunoassay with Siemens Immulite 2000XPi device. The patient was referred to a physician for further evaluation and her physician again requested thyroid function tests with anti-TPO. With advice of his doctor, she was referred to another lab for doing these tests. The results of the second laboratory showed that all of Thyroid function tests were in normal range, so she returned to the first laboratory for consultation. The patient had no previous history of thyroid disease or cervical pain.

The case was re-sampled in the first laboratory and the TSH test result was less than 0.004 IU/ml as before. In the second laboratory the ELISA method was used for measuring serum TSH level. The first laboratory negotiated with the patient for assurance and her sample was sent to a third laboratory for measurement of serum TSH level by other device with Electro Immunoassay method. In the third lab, the Abbott Architect device was used, and again normal range of serum TSH level was taken (Table 1).

**Table 1:** Results of Serum TSH level with different devices and methods in three laboratories

Laboratory	Method	Analyzer device	TSH Result
Al-Mahdi Lab	Chemiluminesance assay	Siemens Immulite 2000XPi	< 0.004 IU/ml
2nd laboratory (Khani abad Lab)	ELISA	Automatic ELISA Human Duo	3.24 IU/ml
3rd laboratory (Danesh Lab)	Electro Immunoassay	Abbott Architect	2.98 IU/ml

Lab manager and physician in Al-Mahdi Clinic explained to the patient about the TSH variant in some people and she was assured that she was not hyperthyroid and doesn’t need any treatment.

## Discussion & conclusion

Serum TSH test is routinely performed in medical laboratories to diagnose and evaluate thyroid gland in patients. Third-generation TSH is the most sensitive one. Immunoassay is currently the method of choice for these tests [1]. However, immunoassays are highly sensitive to variable influences that might lead to false results. False results can be due to the presence of macro-TSH, biotin, Antistreptavidin antibodies, Antiruthenium antibodies and Heterophilic antibodies [2, 3].

Manufacturers of immunoassays kits are trying to reduce the impact of such compounds by adding blocker to the solution or by notifying the user in the brochures. In these cases, clinical records, use of any multivitamin and other medications, history of blood transfusions, autoimmune diseases or direct contact to animals should be considered. When the patient in our study was referred to the laboratory, she had no major disease and did not take any multivitamin containing biotin or other drugs.

She had undetectable serum TSH with normal T3 and T4 levels so she was considered likely to have nodular goiter or other diseases resulting in hyperthyroidism but she had no history of any symptoms of these diseases. The presence of antistreptavidin antibodies, antiruthenium antibodies, and heterophilic antibodies was not considered because these antibodies could also interfere with the results of serum T3 and T4 levels [4].

However, there are other studies which show free T3, freeT4 and T4 levels could be in normal range with increased or decreased serum’s TSH level due to the presence of heterophilic antibodies [5, 6].

Due to the non-detectable TSH by the immunoassay with Siemens Immulite xp2000 and normal-level TSH reported by different other immunoassay devices, she could be considered a TSH variant case.

However, genetic testing should be performed to prove the mutation and these cases should also be investigated for the presence or absence of heterophilic antibodies. In our case, she didn’t consent to further evaluations and she agreed to perform diagnosis tests until her thyroid health was recovered.

Measurement of serum TSH by existing commercial methods of immunoassay is based on the sandwich method in which a robust antibody with a detectable antibody targets in two different regions of TSH. One antibody binds to the beta-TSH and the other one binds to the alpha-beta-TSH. Mutations in the beta-TSH region cause different results in the immunoassays of Siemens and other platforms and this mutation is benign. In our patient, findings were showed the normal bioactivity of variant TSH include clinical euthyroid, normal thyroid hormone levels and normal TSH in assays other than Siemens [7].

Drees JC and his colleagues identified 20 euthyroid patients (19 from South Asia and 1 Iranian) who had undetectable serum TSH level and they diagnosed hyperthyroidism and even began treatment for these patients. Subsequent reassessments revealed that the mutation in the beta-TSH (R55G) region, ie, the conversion of arginine to glycine amino acid, produced different false-negative results on serum TSH levels in different immunoassays. Arginine 55 is a part of the epitope that is identified in immunoassays and its mutation can explain the lack of TSH detection in immunoassays [8, 9].

When the result of serum TSH level is undetectable but the other thyroid function tests are normal range, the TSH test should be repeated by other different methods.

## Data Availability

Not applicable.

## Abbreviations

Anti-TPO: Thyroid-peroxidase antibodies
ELISA: Enzyme-Linked Immunosorbent Assay
T3: Triiodothyronine
T4: Thyroxine
TSH: Thyroid-stimulating hormone

## References

[1] Edith T, Starich GH, Mazzaferri EL (1989). Sensitivity, specificity, and cost effectiveness of the sensitive thyrotropin assay in the diagnosis of thyroid disease in ambulatory patients. Arch Intern Med.

[2] Piketty ML, Polak M, Flechtner I, Gonzales Briceno L, Souberbielle JC (2017). False biochemichal diagnosis of hyperthyroidism in streptavidin-biotin-based immunoassays: the problem of biotin intake and related interferences. Clin Chem Lab Med.

[3] Mogolu S, Armston AE, Mozley E, Nasruddin A (2016). Heterophilic antibody interference affecting multiple hormone assays: is it due to rheumatoid factor?. Scand J Clin Lab Invest.

[4] Favresse J, Burlacu MC, Maiter D, Gruson D (2018). Interferences with thyroid function immunoassays: clinical implications and detection algorithm. Endocr Rev.

[5] Ismail AA, Walker L, Barth H, Lewandowski C, Jones R, Burr A (2002). Wrong biochemistry results: two case reports and observational study in 5310 patients on potentially misleading thyroid-stimulating hormone and gonadotropin immunoassay results. J Clin Chem.

[6] Lewandowski C, Dabrowska K, Lewinski A (2012). Case report: When measured free T4 and free T3 may be misleading. Interference with free thyroid hormones measurements on Roche and Siemens platforms. J Thyroid Res.

[7] Pappa T, Johannesen J, Scherberg N, Torrent M, Dumitrescu A, Refetoff S (2015). A TSHß variant with impaired immunoreactivity but intact biological activity and its clinical implications. Thyroid.

[8] Drees JC, Stone JA, Reamer CR, Arboleda VE, Huang K, Hrynkow J (2014). Falsely undetectable TSH in a cohort of south Asian euthyroid patients. J Clin Endocrinol Metabol.

[9] Rahimkhani M, Einollahi N, Khavari Daneshvar H, Dashti N (2013). Survey of serum procalcitonin in cirrhotic patients. Acta Medica Iranica.

